# Validation and interpretation of Pan-TRK immunohistochemistry: a practical approach and challenges with interpretation

**DOI:** 10.1186/s13000-023-01426-5

**Published:** 2024-01-10

**Authors:** Cansu Karakas, Ellen J. Giampoli, Tanzy Love, David G. Hicks, Moises J. Velez

**Affiliations:** 1 Department of Pathology and Laboratory Medicine, University of Rochester Medical Center, Rochester, NY USA; 2 Department of Biostatistics and Computation Biology, University of Rochester, Rochester, NY USA

**Keywords:** Pan-TRK, *NTRK*, Immunohistochemistry, Interpretation, Validation

## Abstract

**Objectives:**

Actionable, solid tumor activating neurotrophic receptor tyrosine kinase (*NTRK*) fusions are best detected via nucleic acid-based assays, while Pan-TRK immunohistochemistry (IHC) serves as a reasonable screening modality. We describe a practical and cost-effective approach to validate pan-TRK and discuss challenges that may be encountered.

**Methods:**

Pan-TRK Clone EPR17341 was validated in accordance with the 2014 consensus statements set forth by the College of American Pathologists. Confirmation of IHC results were guided by the European Society of Medical Oncology recommendations for standard methods to detect *NTRK* fusions.

**Results:**

Within 36 samples, *ETV6-NTRK3* (n = 8) and *TPM4-NTRK3* (n = 1) fusions were confirmed. *ETV6-NTRK3* fusion positive cases revealed cytoplasmic and nuclear staining. A *TPM4-NTRK3* fusion positive high grade malignant peripheral nerve sheath tumor revealed diffuse cytoplasmic staining. A high grade ovarian serous carcinoma revealed focal punctate staining and revealed a non-actionable *NTRK1* truncation at intron 2. Diffuse cytoplasmic staining was observed in a case of fusion-negative polymorphous adenocarcinoma. Wild-type expression of TRK in pulmonary meningothelial-like nodules was discovered following a false-positive IHC interpretation.

**Conclusion:**

Pan-TRK IHC shows some utility as a diagnostic and surrogate marker for *NTRK* screening however, physiologic or non-specific expression may lead to false-positive results.

## Introduction

Oncogenic *NTRK* fusions are seen in many solid cancer types with a very low incidence and are typically mutually exclusive of other driver mutations. These fusions are particularly important to identify as they are the major determinants for the effectiveness of targeted molecular therapy against these alterations, which makes their routine detection a priority. Neurotrophic tyrosine receptor kinases (*NTRK*) are a family of transmembrane tyrosine kinases that are normally expressed in neural tissue and play a key role in the development and the function of the neural system [1–3]. Three members of this family, *TRKA*, *TRKB*, and *TRKC*, are encoded by the proto-oncogenes *NTRK1*, *NTRK2*, and *NTRK3,* respectively [4, 5]. Each of these proteins are structured with an extracellular ligand-binding domain, a transmembrane region, and an intracellular kinase [4]. In normal conditions, activation of the receptor through ligand binding activates the kinase domain, which leads to receptor homodimerization, phosphorylation and activation of downstream signaling pathways. Constitutive activation of the tropomyosin receptor kinase (TRK) receptors can occur through chromosomal inversions, deletions or translocations that result in an in-frame fusion of the C-terminal tyrosine kinase domain in any of the *NTRK* genes with an N-terminal fusion partner and constitute activation of several cascades or signaling pathways such as phospholipase C, Ras/MAPK/ERK, and PI3K/AKT pathways which promote tumorigenesis [2, 6–8].

Gene fusions involving *NTRK* genes are alterations with known oncogenic potential. Oncogenic fusions between the C-terminal kinase domain with the N-terminal fusion partner of the *NTRK* genes have been identified in high prevalence in rare subtypes of pediatric and adult tumors and are implicated in a small percentage of common adult patient cancers. The first TRK fusion protein was originally described more than 35 years ago in colorectal cancer [9]. In subsequent years, few other studies on *NTRK* gene alterations in colorectal carcinomas have been reported [10, 11]. *ETV6*-*NTRK3* fusion involving a translocation of chromosome 12 and 15 was identified in infantile fibrosarcoma [12–14], and this same fusion was later also reported in both secretory carcinoma of the breast [15, 16] and secretory carcinoma of the salivary gland; previously known as mammary analog secretory carcinoma (MASC) [17]. *NTRK* fusions have also been identified in radiation-induced papillary thyroid carcinomas and in many other tumors, including carcinomas of the lung, gliomas, soft tissue sarcomas, inflammatory myofibroblastic tumors, certain hematologic malignancies, congenital mesoblastic nephroma, and melanocytic tumors, all of which collectively comprise less than 1% of all solid tumors [1, 18–29].

In the last two decades, promising preclinical data on the specific molecular mechanisms of *NTRK* gene fusions resulted in several clinical trials and accelerated the FDA approval of the selective, tumor agnostic *NTRK* inhibitors larotrectinib and entrectinib (pan-TRK, *ROS*1, and *ALK* inhibitor), both of which target a variety of solid tumors in the pediatric and adult populations harboring an *NTRK* fusion [2, 30–35].

These new therapeutic approvals for the treatment of cancers harboring *NTRK* fusions has prompted an urgent need for defining the routine testing criteria to help with clinical decision making and management. Screening for *NTRK* fusions may also be a prerequisite for inclusion into a clinical trial. Furthermore, in uncommon cancers such as infantile fibrosarcoma, congenital mesoblastic nephroma and secretory carcinoma of the breast and salivary gland; identification of *NTRK* fusions may be used to support the diagnosis. Although there are various available assays— such as fluorescence in situ hybridization (FISH), reverse transcription polymerase chain reaction (RT-PCR), DNA-based next-generation sequencing (NGS), and RNA-based NGS, which can detect these fusions at the DNA or RNA level; accurately detecting the variety of *NTRK* fusions with these modalities pose their own challenges and expense, given the limited number of cases that may harbor these alterations (Table 1). For example, DNA-based NGS, may miss fusion detection due to difficulty tiling introns and take several weeks [36]. RNA-based sequencing is advantageous in turnaround time (approximately 1 week) with fusion characterization dependent on RNA quality [36]. Further, the cost of implementation, longer turnaround time, and the need for molecular pathology expertise limits the wide applicable use of sequencing techniques.

**Table 1:** *NTRK1-3* Testing Methodologies

Reverse transcriptase-polymerase chain reaction (RT-PCR)	▪ Variable sensitivity ▪ High specificity	▪ Fusion detection requires specific primers targeting suspected genes and exons ▪ Hindered by RNA degradation
RNA based NGS alone	▪ Ability to access for unknown fusion partners, including other oncogenic alterations as well as splice variants	▪ Hindered by RNA degradation
DNA-based NGS alone	▪ Access for point mutations, fusions, and copy number changes ▪ Limited sensitivity to detect *NTRK3* fusions	▪ Reliant on decent tumor purity
Dual DNA/RNA based NGS	▪ Superior analysis while likely being the most expensive methodology	▪ Reliant on decent tumor purity
Fluorescent in situ hybridization (FISH)	▪ Comparable turn-around time (approximately 1–3 days) compared to IHC ▪ Designed to detect specific breakpoints	▪ Best utilized when there is high suspicion of *ETV6-NTRK3* fusions
Pan-TRK IHC	▪ Ability to screen for *NTRK1-3* fusions while remaining cost effective	▪ Limited specificity ▪ Detects physiologic wild-type TRK expression

In contrast, immunohistochemistry (IHC) is a widely available methodology with several benefits, including quick turnaround time, lower cost and minimal tissue requirements, particularly from limited samples. Nevertheless, there is limited data available regarding the use of Pan-TRK IHC in detecting *NTRK* gene alterations in routine pathology practice [36–41]. Increasing requests for *NTRK* testing by treating physicians, often on limited specimens for advanced or metastatic disease in hopes of identifying an actionable alteration, prompted us to validate and use the pan-TRK diagnostic immunohistochemical assay Ventana pan-TRK (EPR17341) assay as a screening modality for actionable *NTRK* fusions in our laboratory.

The CAP 2014 evidence-based guidelines serve as a practical guide for ensuring accuracy and limiting variation among laboratories validating IHC assays. The CAP authorizes the use of orthogonal testing (non-IHC tests, such as FISH or NGS) to be employed in the validation of an IHC. As stated, “laboratories may use a combination of methods when appropriate” [48]. We present our experience with the validation of pan-TRK IHC in accordance with CAP recommendations with the key aim of making *NTRK* testing easily accessible for laboratories with limited resources. Molecular testing was not indicated on all cases as recommended by the European Society for Medical Oncology practice based guidelines on standard methods for *NTRK* fusion detection in daily practice and clinical research [39].

## Methods

### Design of the Validation Trial

The Ventana pan-TRK assay is a ready-to-use or pre-diluted Food and Drug Administration (FDA) Class I in-vitro diagnostic assay designed to detect the C-region of TRKA, TRKB and TRKC proteins which is conserved in *NTRK* fusions and wild-type proteins [1, 36, 38, 42]. As such, Class I IVD assays, are intended for use in the diagnosis of disease. The pan-TRK assay in this study is not authorized for predictive use or otherwise intended to influence therapeutic decision making alone. Nonetheless, the pan-TRK assay can be utilized as a screening method for NTRK fusions; an actionable, albeit uncommon biomarker for targeted therapy.

For validation, we adhered to the 2014 CAP based guidelines for validation and verification of a non-predictive IHC marker. For validation of a non-predictive IHC assay, the CAP recommends examination of at least 10 positive and 10 negative specimens for initial analytic validation of non-predictive IHC assays with a goal of achieving at least 90% overall concordance between the new test and the comparative test or expected result. Positive and negative controls are required for the validation of an IHC assay. We sought to correlate our assay results (positive or negative expression of pan-TRK IHC) with the expected result based on tumor morphology or tumor diagnosis [48]. We compared our assay result with testing of the same tissue at a reference laboratory with a validated or verified pan-TRK IHC assay; and finally we compared our expected results of the pan-TRK assay with a previously validated or verified non-IHC tests which included fluorescent in-situ hybridization (FISH) using break-apart probes for the detection of *NTRK1, NTRK2* and *NTRK3* genes or confirmation with a molecular assay using an RNA based next-generation sequencing which is limited to targeted regions involving *NTRK1, NTRK3* and *NTRK3* genes. Given the pan-TRK IHC is not authorized as a predictive biomarker, guidance on when confirmatory testing for *NTRK* fusions should be performed were adapted by recommendations set forth by the European Society of Medical Oncology (ESMO) on the standard methods to detect *NTRK* fusions [39].

This validation protocol is not designed to study the predictive nature of the pan-TRK IHC to identify *NTRK* fusions; therefore, based on ESMO recommendations, orthogonal testing for the presence of a pan-TRK fusion was not performed on each individual case. This protocol is specifically intended for the validation of pan-TRK IHC for clinical use while remaining cost-effective.

### Pan-TRK IHC Staining Procedure

IHC staining for pan-TRK expression was performed with iVIEW DAB Detection Kit (Ventana Medical Systems, Tucson, AZ), using a commercially available pan-TRK assay (predilute rabbit monoclonal antibody, clone EPR17341, Assay, RTU, Roche, Ventana). Tissue slides were produced from surgical pathology and cytopathology formalin-fixed, paraffin-embedded (FFPE) neoplastic tissue samples. Tissue slides were pretreated with a high PH heat-induced epitope retrieval (CC1) for 88 min and endured a 16-min antibody incubation time. Immunohistochemical stains were performed on the automated Benchmark Ultra platform (Ventana Medical Systems, Tucson, AZ). All assays were performed in a CLIA certified- laboratory.

Normal appendix tissue was used as control which showed weak or strong membranous and cytoplasmic staining of nerves and ganglion cells and negative staining in mucosa, smooth muscle, lymphoid aggregates and glandular epithelium, all of which served as positive and negative internal controls.

### Sample selection

Rare tumors which commonly harbor *NTRK* fusions were considered as the gold standard for the positive control group while tumors which uncommonly harbor *NTRK* fusions were considered for the negative control group. Relatively common tumors which infrequently harbor *NTRK* fusions and may be considered include non-small cell lung cancer, colorectal carcinoma, pancreatic adenocarcinoma, cutaneous melanoma, and invasive breast carcinoma [49].

We selected a total of 36 tumors diagnosed from 2015 to 2020. Clinicopathologic characteristics of the patients are represented on Table 2. Selected cases included 5 cytology and 31 surgical specimens. For the positive control group, we identified cases of secretory carcinoma of the breast, and secretory carcinoma of the salivary gland, including its historical synonym of mammary analogue secretory carcinoma. Secretory breast carcinoma and secretory carcinoma of the salivary gland harbor *NTRK* fusions in up to 92.78% and 79.86%, respectively [53].

**Table 2:** Clinicopathological characteristics of 36 patients evaluated for Pan-TRK expression

**PATIENT CHARACTERISTICS**	**No**		**%**
Age range	46–88 y		
Median	67.5		
Female	19		52.8
Male	17		47.2
**HISTOLOGIC TYPE & SOURCE**		**SITE OF ORIGIN**	
**Fusion positive cases (*n* = 9)**			
Secretory carcinoma of the salivary gland (MASC)	6	Parotid/salivary gland	16.8
Secretory carcinoma	1	Breast	2.8
Metastatic MEC, lung	1	Parotid	2.8
MPNST	1	Soft tissue	2.8
**Fusion negative cases (*n* = 26)**			
Invasive carcinoma	1	Breast	2.8
SMARCA4-BRG-1-deficient neoplasm	1	Lung	2.8
Adenocarcinoma, lung	9	Lung	25.2
Adenocarcinoma, lung	2	Colorectal (*n* = 1), unknown origin (*n* = 1)	5.6
Polymorphous adenocarcinoma	1	Soft palate	2.8
Adenocarcinoma, lymph node	5	Lung (*n* = 4), urothelial (*n* = 1)	14
Adenocarcinoma, pericardial fluid	1	Lung	2.8
Adenocarcinoma, pleural fluid	2	Lung (*n* = 1), unknown origin (*n* = 1)	5.6
Adenocarcinoma, soft tissue	1	Lung	2.8
Adenocarcinoma, bone	2	Lung (*n* = 1), unknown origin (*n* = 1)	5.6
High grade serous carcinoma	1	Ovary	2.8
**Fusion status N/A (*n* = 1)**			
Adenocarcinoma, pleura	1	Lung	2.8

*Abbreviations***:** *MASC* Mammary analog secretory carcinoma, *MPNST* Malignant peripheral nerve sheath tumor, *HGSC* High-grade serous carcinoma, *N/A* Not available

We did not identify cases of infantile fibrosarcoma or congenital mesoblastic nephroma which reportedly harbor *ETV6-NTRK3* in approximately 90% of cases [51–53]. A various collection of tumors types which uncommonly harbor *NTRK* fusions were used for negative control specimens however, primarily consisted of lung adenocarcinoma given its exceedingly low incidence of *NTRK* fusions, which may be detected in less than 1% of cases [51]. Lastly, pathologic reports including addenda were searched for reported “*NTRK*” fusion results or other alterations. In general, neuroendocrine tumor and gliomas were not used given the cross-reactivity with normal or wild-type expression of TRKA, TRKB and TRKC proteins.

The tumor types and sites examined in our validation are summarized in Table 2. Hematoxylin and eosin (H&E)-stained sections were evaluated and the diagnoses were confirmed, including examination by a Head and Neck Pathologist for salivary gland tumors.

### Pan-TRK scoring

Pan-TRK expression was considered positive if the following subcellular staining patterns of any intensity were observed in ≥ 1% of tumor cells [1, 36, 40, 42–44]. Staining intensity was graded as negative (0), weak (1 +), moderate (2 +) or strong (3 +). Cytoplasmic and nuclear reactivity was considered positive for expression. Punctate staining alone was considered non-specific or equivocal.

### Pan-TRK IHC validation

For cases in our negative control group, internal interpretation of pan-TRK results were compared to an outside interpretation performed at a reference laboratory offering a pan-TRK assay which is validated for clinical use. As per ESMO recommendations, for tumors which uncommonly harbor *NTRK* fusions, we did not pursue RNA-based sequencing for negative IHC results [39]. Additionally, in accordance with CAP guidelines, results of our negative control group were compared with the expected results based on tumor morphology (i.e. the negative control group is expected to yield a negative result based on the tumor diagnosis and low incidence of pan-TRK fusions in the tested tumor type).

In keeping with ESMO recommendations, in tumors suspected to harbor *NTRK* fusions or in cases with positive pan-TRK IHC expression and no prior confirmatory testing, results were confirmed by RNA based next-generation sequencing performed at an outside reference laboratory for confirmation of the fusion transcript. However, RNA-based sequencing was also attempted on equivocal IHC results. Non-concordant IHC results were recorded as a discrepancy. Lastly, we referred to preexisting or reported RNA sequencing and FISH results, confirming the presence of an *NTRK* fusion in select cases as identified in the archives.

### Pan-TRK IHC results and interpretation

All cases (n = 36) were tested by pan-TRK IHC which included 9 *NTRK3-*fusion positive cases (8 cases with *ETV6-NTRK3* fusion and 1 case with *TPM4-NTRK3* fusion).

**Fig. 1:**
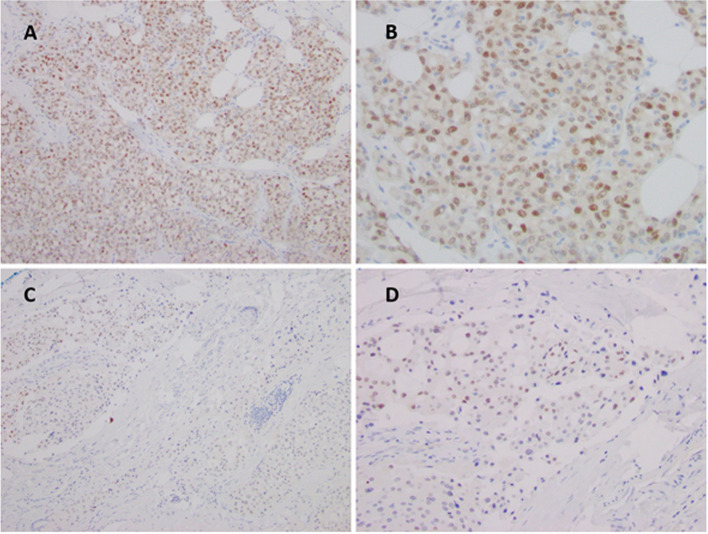
Different patterns of pan-TRK immunostaining with proven *ETV6-NTRK3* fusion cases. A-B, Secretory carcinoma of the breast with diffuse nuclear and cytoplasmic staining. C-D, Focal and weak expression of Pan-TRK staining in secretory carcinoma of the parotid gland (Original magnification × 200, insets × 400)

**Fig. 2:**
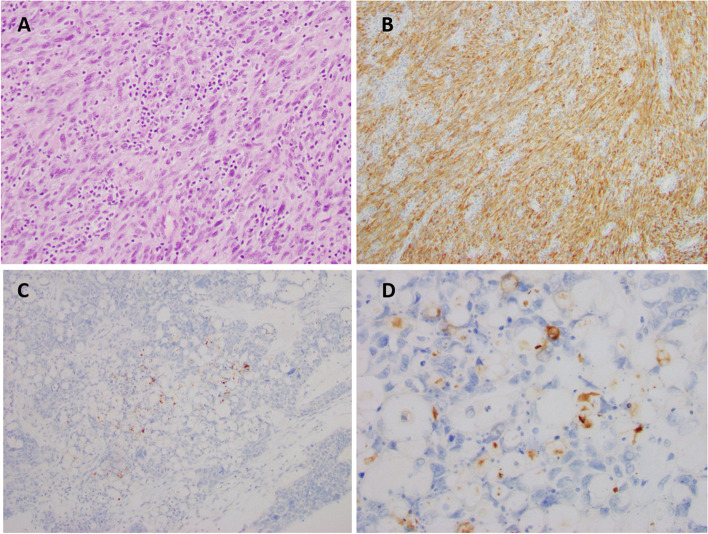
Cases with various *NTRK* gene alterations. A, Representative H&E image of malignant peripheral nerve sheath tumor (MPNST). B, Strong membranous staining in a MPSNT harboring *TPM4-NTRK3* fusion (Original magnification × 200, inset × 400). C-D, High grade ovarian serous carcinoma with focal punctate staining in areas with a microcystic pattern. This case revealed a *NTRK1* truncation at intron 2 on NGS testing (Original magnification × 200, inset × 400)

In the *ETV6-NTRK3* fusion cohort, the pattern of Pan-TRK staining was both cytoplasmic and/or nuclear in all 8 out of 8 cases (Fig. 1A-B). The tissue distributions of cancers with *ETV6-NTRK3* fusions included mammary analog secretory carcinoma (*n* = 6), metastatic mucoepidermoid carcinoma (*n* = 1), and breast secretory carcinoma (*n* = 1) and malignant peripheral nerve sheath tumor (*n* = 1) (Table 3). In two *ETV6-NTRK3* fusion positive cases with Pan-TRK expression, staining was minimal (in approximately 5% of cells) (Fig. 1C-D). Of note, in three resection cases, nuclear staining was observed more at the periphery of the tumors and may be suggestive of a problem with adequate tissue fixation. One *TPM4-NTRK3* fusion positive high grade malignant peripheral nerve sheath tumor (HGMPNST) revealed diffuse strong (3 +) cytoplasmic staining only. (Fig. 2A-B).

**Table 3:** Malignancies with Positive or equivocal (*n*=12) Internal (in-house) Pan-TRK IHC expression and *NTRK *NGS results

**Tumor location**	**Tumor diagnosis **	***NTRK *NGS**	**Pan-TRK IHC ** **Reference lab**	**Pan-TRK IHC** **In-house**
Parotid gland	Secretory carcinoma	*ETV6-NTRK3*	Positive	Positive (nuclear, 2+, 5%)
Parotid gland	Secretory carcinoma	*ETV6-NTRK3*	Positive	Positive (cytoplasmic, 1+, 20%)
Parotid gland	Secretory carcinoma	*ETV6-NTRK3*	Positive	Positive (cytoplasmic 1+, 100%; rare, focal nuclear staining, 2+, peripherally)
Parotid gland	Secretory carcinoma	*ETV6-NTRK3*	Positive	Positive (cytoplasmic, peripherally, 1+, 5%)
Parotid gland	Secretory carcinoma	*ETV6-NTRK3*	Positive	Positive (nuclear, peripherally, 2+, 15%)
Submaxillary gland	Secretory carcinoma	*ETV6-NTRK3*	Positive	Positive (cytoplasmic 1+, 100%; scattered nuclear, 2+)
Lung	Metastatic MEC	*ETV6-NTRK3*	Positive	Positive (cytoplasmic and nuclear staining, 1-2+, 100%)
Breast	Secretory carcinoma	*ETV6-NTRK3*	Positive	Positive (nuclear and cytoplasmic, 2-3+, 70%)
Soft tissue-popliteal area	MPNST	*TPM4-NTRK3*	Positive	Positive (cytoplasmic, 3+, 80%)
Soft palate	Polymorphous adenocarcinoma	No fusion detected	Positive	Positive (cytoplasmic, 2+, 90%)
Pleural fluid	Metastatic carcinoma, unknown origin	N/A	Positive, Focal	Equivocal (punctate, 1%)
Ovary	HGSC	*NTRK1 *truncation- intron 2	Positive, Focal	Equivocal (punctate, 5%)

**Abbreviations:** *MPNST* malignant peripheral nerve sheath tumor, *HGSC* high-grade serous carcinoma, *MEC* mucoepidermoid carcinoma, *N/A* not tested

Notably, focal or scattered nuclear staining was observed in 2 out of 8 *ETV6-NTRK3* positive cases. Recently published studies showed that the staining patterns of the Pan-TRK antibody can vary in intensity and localization. Nuclear staining has been described in tumors harboring *NTRK3* fusions, which can be used as a surrogate for the presence of *NTRK3* fusions; however, the staining in these tumors can be weak and may cause false negative results [36, 37, 42, 43]. This weak focal staining may be responsible for a reduced sensitivity rate for *NTRK3* fusions compared to fusions involving *NTRK1* and *NTRK2* [1, 42]. Other staining pattern described with *ETV6-NTRK3* fusions include cellular membrane staining, cytoplasmic staining, and nuclear expression with accentuation of the nuclear envelope [28, 36, 40, 42]. All our cases with pan-TRK expression showed cytoplasmic staining, which was consistent with a prior assessment conducted by Hechtman et. al. using the same clone, EPR17341 [36] The staining patterns observed correlate with the subcellular localization of the most common *NTRK* fusion partners, *ETV6* and *TPM4*.

Our analysis of polymorphous adenocarcinoma (*n* = 1) showed diffuse moderate cytoplasmic expression with pan-TRK IHC, both in-house and at the reference laboratory. While the IHC interpretation is concordant, orthogonal testing with RNA based NGS was negative for *NTRK1-3* gene fusions.

We interpreted two cases as equivocal. One case of metastatic adenocarcinoma in the pleural fluid demonstrated granular cytoplasmic staining in scattered tumor cells and interpreted as equivocal staining internally and positive at the reference laboratory however; there was insufficient tissue for further orthogonal testing with RNA based NGS or FISH. Similarly, a discordant case of high grade ovarian serous carcinoma was interpreted as equivocal staining internally and interpreted as focally positive at a reference laboratory (Table 3). This case showed focal punctate staining in areas of tumor cells with a microcystic pattern (Fig. 2, C-D), which to our knowledge has not been described. Orthogonal testing revealed a *NTRK1* truncation at intron 2 when reflexed to NGS *NTRK* gene fusion analysis at a reference laboratory. Although *NTRK1* gene alterations in gynecologic tumors are rare, a recent case report presented a recurrent ovarian cancer with *NTRK1-TPM3* fusion, in which entrectinib was ineffective. In this case report, *NTRK1* gene fusion was detected by DNA-based NGS and IHC using Clone EPR17341 (Abcam, Cambridge, MA) was negative however; a scoring system for the interpretation of pan-TRK IHC was not described [47]. In contrast, cytoplasmic staining identified in tumors harboring *NTRK1* fusions have been reported with different intensities.[1, 40, 41, 43].

Twenty-five tumors that uncommonly harbor *NTRK* fusions were interpreted as negative for pan-TRK expression internally and at the reference laboratory (Table 4). A discordant case of metastatic adenocarcinoma, favored to be of urothelial origin was interpreted as negative internally and focally positive at the reference laboratory. Similarly, a discordant case of pulmonary adenocarcinoma sampled by fine needle aspiration, was interpreted as negative for pan-TRK internally and as equivocal (focal) at the reference laboratory. Orthogonal testing was not pursued in these two cases given the tumor subtypes or unlikelihood of harboring an *NTRK* fusion and unequivocally negative internal result.

**Table 4:** Malignancies with Negative (*n* = 24) Internal (in-house) results Pan-TRK IHC expression and *NTRK* results

Tumor location	Tumor diagnosis	*NTRK* analysis	Pan-TRK IHC Reference lab	Pan-TRK IHC In-house
Breast (*n* = 1)	Invasive carcinoma, exclude secretory carcinoma	*ETV6-NTRK3* FISH negative	-	Negative
Lung (*n* = 1)	BRG-1 deficient tumor	-	Negative	Negative
Lung (*n* = 7)	Pulmonary adenocarcinoma	*-*	Negative	Negative
Pericardial fluid (*n* = 1)	Pulmonary adenocarcinoma	*-*	Negative	Negative
Lymph node (*n* = 1)	Adenocarcinoma with Neuroendocrine differentiation	*-*	Negative	Negative
Lymph node (*n* = 2)	Pulmonary adenocarcinoma	*-*	Negative	Negative
Lung (*n* = 1)	Colorectal adenocarcinoma	-	Negative	Negative
Bone (*n* = 1)	Poorly differentiated carcinoma	-	Negative	Negative
Bone (*n* = 1)	Pulmonary Adenocarcinoma	-	Negative	Negative
Soft tissue (*n* = 1)	Pulmonary mucinous adenocarcinoma	*-*	Negative	Negative
Lymph node (*n* = 1)	Pulmonary adenocarcinoma	-	Negative	Negative
Lymph node (*n* = 1)	Urothelial adenocarcinoma	-	Negative	Negative
Lung (*n* = 1)	Adenocarcinoma, unknown origin	-	Negative	Negative
Pleural fluid (*n* = 2)	Pulmonary adenocarcinoma	-	Negative	Negative
Lung, FNA (*n* = 1)	Pulmonary adenocarcinoma	-	Equivocal (focal)	Negative
Lung (*n* = 1)	Adenocarcinoma, Meningothelial-like nodule	Negative for *NTRK1-3* fusion by NGS	Positive	Negative

**Fig. 3:**
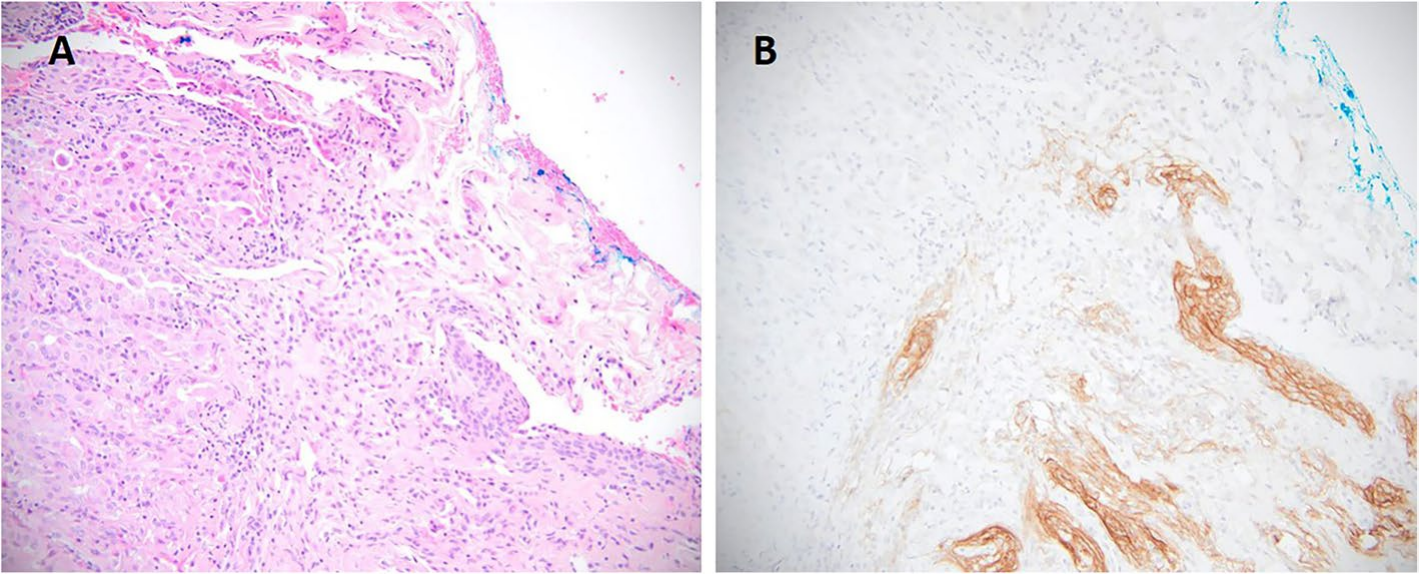
Pan-TRK immunostaining in a meningothelial-like nodule (MLN). A, Representative H&E image of the meningothelial-like nodule located adjacent to the primary tumor in a lung resection specimen leading a false positive result. B, MLN shows strong cytoplasmic and membranous pan-TRK expression (original magnification × 200)

Retrospective review of H&E sections of a discordant case of lung adenocarcinoma which was interpreted as negative internally and positive at the reference laboratory, showed an adjacent entrapped meningothelial–like nodule (MLN) which exhibited strong, diffuse pan-TRK expression (Fig. 3, A-B). Co-occurrence of lesions with neural differentiation such as MLNs near or entrapped within the tumor bed, can make the interpretation of Pan-TRK IHC challenging. To distinguish between wild-type pan-TRK protein expression versus the presence of an *NTRK* fusion in MLNs, we expanded our analysis and performed RNA sequencing for *NTRK* fusions on 8 additional cases of MLNs identified from our archives. Out of 8 cases, 3 cases were excluded following microdissection due to insufficient lesional material being left for sequencing. 4 out of 5 of remaining cases had multiple MLNs on one section. All remaining 5 cases (100%) showed diffuse positive pan-TRK expression without confirmed *NTRK* fusions. Our findings therefore support wild-type expression of pan-TRK proteins in MLNs. Fusion analysis of MLNs was an important endeavor to distinguish between the potential identification of an *NTRK* fusion following orthogonal testing. Fortunately, a false positive pan-TRK result in the setting of MLNs will not lead to therapeutic implications as the presence of *NTRK* fusions in MLNs has not been identified.

We were able to access the sensitivity and specificity based on any pan-TRK interpretation, and correlation with confirmatory testing results (Tables 3 and 4). Albeit a major limitation in accessing the predictability of this assay, NGS was not performed on all cases to make this a cost effective clinical validation. This was not intended to study the predictive correlation of pan-TRK expression and orthogonal testing results. As previously stated, per ESMO recommendations, orthogonal testing for the presence of *NTRK* fusion was not performed on negative cases given the exceedingly low probability of harboring a fusion based on specific tumor subtypes. In total, pan-TRK IHC was performed on 36 tumors and of those, 13 cases were interpreted as positive in-house or at the reference laboratory. One case with positive pan-TRK expression (pleural fluid) had insufficient tumor material for orthogonal testing. Therefore, NGS testing was performed on 12 of 13 cases.

Of these 12 pan-TRK positive cases, *NTRK* fusions were identified in 9 cases (true positives), while (following ESMO guidelines), testing for *NTRK* fusions was not performed on the presumed negative cohort (presumably, 0 false negatives). Therefore, the combined sensitivity of the pan-TRK IHC assay to identify *NTRK* fusions is 100%. Of the 26 expected and presumed fusion negative cases, pan/TRK IHC was positive in 3 cases (false positives). The combined specificity of the pan-TRK assay is 88%.

Among the tumors with pan-TRK expression and molecular testing, *NTRK3* fusions (true positives) were identified with 100% sensitivity in 7/7 secretory carcinomas, 1/1 mucoepidermoid carcinoma and 1/1 malignant peripheral nerve sheath tumor.

Among the expected and presumed negative cohort (n = 26), false positive pan-TRK expression was observed in polymorphous adenocarcinoma (1/1), high grade ovarian serious carcinoma (1/1) and a single case of lung adenocarcinoma harboring a meningothelial-like nodule leading to false impression of epithelial cell staining.

Overall, there was 89% (32/36) concordance for the validation study between the pan-TRK IHC results conducted in-house and the reference laboratory including results for pan-TRK IHC, *NTRK* FISH, and RNA based *NTRK* 1–3 NGS fusion analysis used as a comparison (Tables 3 and 4). Interobserver agreement for pan-TRK IHC results between in the in-house pathologist and reference laboratory was observed; showing a Kappa coefficient of 0.63 (*p* < 0.001).

In conclusion, our overall concordance for our assay was slightly below the required parameters for the CAP recommendation of at least 90% overall concordance with expected results when compared to all comparator test i.e. outside reference laboratory and expected *NTRK* fusion positive results. Per CAP guidelines, if a laboratory director determines fewer validation cases are sufficient for a particular marker (such as for a rare antigen or tissue), the defendable rationale must be recorded in the validation summary. Given the rarity of tumors which harbor *NTRK* fusions, expected positive cases are difficult to come by, making meeting all CAP IHC validation parameters challenging, despite the relatively low number of minimum cases recommended for non-predictive markers (10 positive and 10 negative cases) and ease of use encountered with an in-vitro diagnostic assay. We therefore determined, the fewer number of positive controls were adequate for the validation, especially given our limited population showed positive pan-TRK expression in all (100%) *NTRK* rearranged tumors. Our results indicate that pan-TRK IHC is limited as a diagnostic marker however, has some utility as a marker to help screen for the identification of possible *NTRK* fusions; particularly *ETV6-NTRK3* however, only if nuclear expression is observed and if an *NTRK* fusion is expected based on the tumor subtype.

## Discussion

Pan-TRK IHC is a widely available, technically less challenging to perform and interpret, and may provide clinically actionable and time efficient results; especially if urgent therapeutic intervention is required, while awaiting orthogonal confirmation. Outside of major academic institutions with expanded fusion panels that may account for the detection of *NTRK1-3* gene alterations, the vast majority of *NTRK* mutations may go undetected in advanced or metastatic disease. Detection of *NTRK* fusions in advanced or metastatic cases are recommended by National Comprehensive Cancer Network (NCCN) guidelines for cancers of lung, colon, breast, central nervous system cancers, pancreatic, gastrointestinal, hepatobiliary, thyroid and ovarian origin [50]. Validating Pan-TRK IHC may otherwise bridge this gap or delay in *NTRK* testing, especially if pan-TRK IHC is employed at the time of pathologic evaluation of advanced or metastatic cancers.

Evaluation of pan-TRK IHC is not without its challenges and may not be suitable for screening in all NCCN recommended cancers. A retrospective review of samples (2018–2021) to identify a correlation between positive pan-TRK (clone EPR17341) results and the presence of an *NTRK* gene fusion by Koehne de González et al., highlights the difficulty or lack of specificity when pan-TRK IHC is employed indiscriminately as well as the importance of NGS as the gold standard for detection of *NTRK* fusions. Koehne de González et al. reported a limited sensitivity to detect fusions however; 20/63 (31.7%) of cases with positive pan-TRK expression showed neuroendocrine or myogenic differentiation which are likely confounded by wild-type expression. Furthermore, weak or focal staining likely due to wild-type or non-specific expression was noted in 71.4% of cases, which did not include tumor types with a high incidence of *NTRK* fusions [50]. RNA fusion panel analysis of 127 cases identified 4 cases harboring *NTRK* fusions. Strong membranous and cytoplasmic pan-TRK IHC staining was reported in a case of *TPM3-NTRK1* fusion positive colorectal carcinoma. Equivocal staining was noted in a pancreatic adenocarcinoma harboring a *GOLGA4-NTRK3* fusion in which granular staining or “ISH-Like” staining was described. As an example of IHCs limited sensitivity, two cases (primary and metastatic tumors of lung) harboring *ADAM19-NTRK3* fusions were negative for pan-TRK IHC. Per their institutional clinical practice, not all pan-TRK negative cases were accessed by RNA sequencing therefore the sensitivity could not be accessed [50]. As a general recommendation, the use of pan-TRK IHC as a screening modality should be limited in tumors with the potential of physiologic expression, such as gastrointestinal stromal tumors, neuroblastomas, glioblastomas and leiomyosarcomas [51].

A recent study by Mohamed et al. accessing the diagnostic value of pan-TRK (clone EPR17341, AbCam) in the detection of *NTRK* fusions in a subset (*n* = 23) of central nervous system (CNS) tumors, detected expression in 11/23 (47.8%) tumors. 2/11 tumors (liponeurocytoma and gliobastoma) with pan-TRK expression harbored *AGBL4-NTRK*, *BEND5-NTRK2* fusions while no fusions were detected by NGS in all 12 tumors without pan-TRK expression (5). In the CNS, this study illustrates the inability of pan-TRK to distinguish between physiologic *NTRK* expression and the presence of an *NTRK* fusion however, the lack of staining can confidently exclude the latter.

The most common staining pattern encountered is cytoplasmic reactivity which can be observed in fusion positive and physiologic expression which can lead to a false-positive interpretation [51]. Given the conundrum of focal and weak staining which can be detected with pan-TRK IHC and the established lower limit of positivity (i.e. positivity in at least 1% of the tumor cell population), knowledge of the incidence of *NTRK* fusions in the tumor subtypes may influence whether such staining is interpreted as non-specific or equivocal. For example, *NTRK* fusions are detected in < 1% of NSCLCs, therefore, any increase in the frequency of staining in surgical or cytology specimens (after confirming a lack of background staining in the control tissue), should likely be regarded as equivocal and followed by reflexive testing with an orthogonal method. Since even NGS sequencing can be susceptible to a false negative result for *NTRK* fusion detection, your diagnostic consideration and insight into the incidence in *NTRK* fusions, such as the high incidence of *NTRK* fusions in secretory carcinoma of the salivary gland and breast, should prompt a conversation regarding the methodology or limitation of the NGS assay [51].

It’s important to note, false-positive staining has been described in approximately 8–10% of secretory carcinoma of the breast and salivary gland [51]. Among initial studies accessing pan-TRK IHC, Hechtman et al. reported a sensitivity and specificity of 95.2% and 100%, respectively and Rudzinski et al. reported a sensitivity and specificity or 97% and 98%, respectively, both of whom evaluated pan-TRK clone EPR17341 for the detection of *NTRK* rearrangements. Recently published studies additionally have shown sensitivities ranging from 75 to 92.5% and specificities between 81.1 and 100% [1, 36, 42, 45].

As a consideration, commercially available non-IVD pan-TRK IHC clones may be validated as a laboratory developed test. While the CAP guidelines for validation of an IHC antibody does serve to reduce variation amongst CAP accredited laboratories within the United States and its territories, a universal support plan for implementation of the pan-TRK assay does not exist, likely given the challenges posed, such as the implementation across various IHC platforms and validation as a laboratory developed test (for non-IVD antibodies).

In contrast, the Canadian CANTRK Ring Study, developed as a multicenter collaboration, underwent the endeavor of assisting laboratories across Canada to validate laboratory-developed IHC assays for pan-TRK screening, including the validation of NGS for the detection of *NTRK* fusions. For the adult population, the Canadian consensus group focused on methodologies in the detection of *NTRK* fusions in five specific neoplastic subgroups, which included thyroid carcinoma, colorectal carcinoma, NSCLC, soft tissue sarcomas and salivary gland carcinoma. In contrast to the ESMO guidelines which recommends a tumor agnostic approach based on the likelihood of harboring an *NTRK* fusions, the Canadian consensus group described recommendations for NGS testing dependent on the cancer subtype, extent of disease and provides a discussion on around available therapeutic options. While a separate publication on the Canadian consensus for *NTRK* biomarker testing in the pediatric populations exists, we intend to summarize key recommendations for the adult population by the Canadian consensus group on the use of pan-TRK IHC versus NGS analysis for *NTRK1-3* genes, which may serve as a guide to direct subspecialty testing for *NTRK* alterations [53, 56].

TRK fusion positive thyroid carcinomas are clinically aggressive with high metastatic rates and multinodular growth [53]. For unresectable, metastatic or advanced thyroid cancer, pan-TRK IHC screening is recommended to be performed on all cases followed by NGS testing for *NTRK* to confirm IHC positive and equivocal cases [53].

For colorectal carcinoma, *NTRK* fusions are typically mutually exclusive of other mutations, thus the ideal scenario includes reflexive *NTRK* testing via NGS in all *RAS/BRAF* V600E wild-type, microsatellite instability (MSI)-high and mismatch repair deficient (dMMR) [53]. This strategy results in < 5% of metastatic patients requiring *NTRK* fusion testing; however, account for up to 90% of detected *NTRK* fusion positive colorectal carcinomas [53]. Further, since 11–23% of TRK fusion positive colorectal carcinomas have been identified in microsatellite stable/ MMR proficient (pMMR) tumors; it reasonable to consider *NTRK* gene fusion testing in MSI stable/ pMMR, *RAS/ BRAF* V600E wild-type patients however; testing this population accounts for 40% of cases tested [53]. If resources do not allow *NTRK1-3* gene fusion testing, pan-TRK IHC is a suitable alternative with NGS testing performed to confirm a positive result.

The incidence of *NTRK* fusions in NSCLC is extremely low (0.23%), with a predilection to occur in younger patients (Median age of 47.6 years) with a 0–5-year smoking history. This specific demographic accounts for 73% of *NTRK* gene fusions in NSCLC with the vast majority of fusions detected in pulmonary adenocarcinoma (82%) [21, 53]. Since *NTRK* fusions are considered to be mutually exclusive, *NTRK* gene fusion testing should be ideally be considered as part of a larger molecular panel testing for *ALK*, *ROS1*, *EGFR*, *KRAS*, *RET*, *BRAF*, *ERBB2* exon 20, *NRG1* and *MET*. The Canadian consensus recommends integration of routine testing for *NTRK*1-3 genes in Stage III/IV patients with locally advanced or metastatic disease, in patients with non-squamous or adenocarcinoma components. *NTRK* testing can be considered for squamous cell carcinoma with mixed histology (example adenosquamous carcinoma) or in patients with a light smoking history. For limited molecular panels, the use of pan-TRK IHC is permissible, with or without *NTRK* fusion testing to confirm wild-type patients in non-squamous NSCLC. If pan-TRK IHC is performed after sequential molecular testing to first exclude the possibility of more common driver mutations, pan-TRK IHC testing would then be limited to 65% of NSCLC cases.

Within the vast soft tissue tumor differentials, inflammatory myofibroblastic tumor-like, fibrosarcoma/ malignant peripheral nerve sheath tumor-like or lipofibromatosis-like morphologic patterns show a higher incidence of harboring *NTRK* fusions [53]. Currently, *NTRK* testing is not recommended in leiomyosarcoma or liposarcoma since *NTRK* fusions have not been identified to date. *NTRK* fusions are mutually exclusive and are uncommonly identified in a subset of *KIT/PDGFRA* wild-type gastrointestinal stromal tumors (GIST). In this setting, *NTRK1-3* gene fusion and *BRAF* mutation testing via NGS is recommended [53]. Otherwise, pan-TRK IHC may be utilized on other locally advanced or metastatic non-GIST soft tissue tumors with are negative for known or diagnostic molecular alterations to screen for *NTRK* fusions; followed by NGS for positive of IHC equivocal results [53].

Secretory carcinoma of the salivary gland accounts for 4–5% of salivary gland carcinomas, with the vast majority of them harboring *NTRK3-ETV6* gene fusions. NGS is recommended for actionable *NTRK* and *RET* gene fusions [53]. A broad molecular panel is recommended if there is unclear histology. However, for all other salivary gland carcinomas, NGS is recommended in only locally advanced or metastatic disease [53]. *NTRK* testing may also be pursed following androgen receptor and *HER2* IHC analysis [53]. Given the low incidence of salivary gland carcinomas, pan-TRK IHC is not recommended by the Canadian consensus group however, it may be considered to conserve resources and recommends confirmatory NGS or other molecular test following a positive or equivocal IHC result.

In summary, the Canadian consensus for biomarker testing and treatment of TRK fusion cancers offers a practical approach to testing for *NTRK* fusions, including the implementation of pan-TRK IHC; and to our knowledge remains the only expert consensus in North America to guide testing in the adult population to-date. While ultimately, the optimal testing strategy may be guided by the availability of institutional assays and expertise, comprehensive NGS remains the gold standard for detection of *NTRK* fusions and other targetable alterations across multiple tumor types.

At our institution, clinically advanced or metastatic cases are typically sent for RNA NGS to test for various alterations for targeted therapy or clinical trial inclusion while pan-TRK IHC serves to supplement initial analysis of a limited molecular panel. If NGS is not routinely performed in advanced malignant tumors, most algorithms support using pan-TRK IHC as screening method following by confirmation of positive cases with an orthogonal method, while acknowledging feasibility and cost should be taken into consideration [51].

There are benefits and limitations to consider with other testing methodologies utilized for the confirmation of *NTRK* fusions. Reverse transcriptase-polymerase chain reaction (RT-PCR) for example, shows variable sensitivity and high specificity however, detection of fusions requires specific primers targeting the involved or suspected genes and exons. RNA based NGS, like RT-PCR, may be hindered by RNA degradation however, provides the benefit of assessing for unknown fusion partners across multiple gene types, including other oncogenic alterations as well as splice variants [54]. While DNA-based NGS alone may access for point mutations, fusions, and copy number changes, the sensitivity for *NTRK3* is limited (76.9%) and is reliant on decent tumor purity. Dual DNA/RNA based NGS offers superior analysis while likely being the most expensive methodology. FISH results offer a comparable turn-around time (approximately 1–3 days) compared to IHC however, FISH is designed to detect specific breakpoints and is likely best utilized when there is high suspicion of *ETV6-NTRK3* fusions [54]. IHC, while limited in specificity, displays the ability to detect *NTRK1-3* fusions, and wild-type TRK expression (54).

To date, only *NTRK1-3* gene fusions have been identified as targetable alterations responsive to TRK inhibitors [54]. In-vitro analysis of tumor cells harboring known *NTRK* point mutations show no tumor driver potential but demonstrated impaired receptor activation and downstream signaling of no functional difference from wild-type receptors [54]. TRKB overexpression in neuroblastoma was associated with higher grade tumors with preclinical studies demonstrating responsiveness to TRK inhibitors in cells lines. Furthermore, high expression of full-length TRKC (*NTRK3*) in a *EWSR1-WT1* fusion positive desmoplastic small round cell tumor showed sensitivity to TRK inhibitor therapy [54]. Diffuse membranous and cytoplasmic staining with *NTRK3* IHC in DSRCT was identified confirming wild-type protein expression. In this study, two *NTRK3*-null DSRCT cell lines showed reduced tumor viability, supporting inhibition of *NTRK3* by entrectinib and reprotrectinib. This study remarkably shows the transcriptional activation of *NTRK3* by *EWSR1-WT1* plays a significant role in the proliferation of DSRCT. This example highlights that further studies are needed to elucidate the functional significance of wild-type *NTRK* expression in tumor subtypes with known alterations if identified with pan-TRK IHC [53, 54].

We hope our experience and discussion will provide guidance into the validation, interpretation and implementation of pan-TRK IHC while creating awareness of potential pitfalls and challenges with interpretation. While further literature contributions and expert consensus from the Association for Molecular Pathology (AMP), may later define a standardized role for pan-TRK-IHC, for the foreseeable future it seems, pan-TRK IHC will continue to have a role as a screening method for actionable *NTRK* fusions until other testing methodologies continue to become widely available. Establishing an optimal algorithm for the detection of *NTRK* fusions may be dependent on the tumor subtype and perhaps best established by subspecialty consensus when possible. The goal of screening with immunohistochemistry or other methodology is intended to prolong patient survival by identifying patients who may benefit from TRK inhibitors, in a population that would otherwise go undetected.

## Data Availability

All data supporting the findings and conclusions of this study is included within the article.
